# Literature Review of Prognostic Factors in Secondary Generalized Peritonitis

**DOI:** 10.3390/life15060880

**Published:** 2025-05-29

**Authors:** Valerii Luțenco, Adrian Beznea, Raul Mihailov, George Țocu, Verginia Luțenco, Oana Mariana Mihailov, Mihaela Patriciu, Grigore Pascaru, Liliana Baroiu

**Affiliations:** 1 Faculty of Medicine and Pharmacy, “Dunărea de Jos” University of Galati, 800008 Galați, Romania; valerii.lutenco@ugal.ro (V.L.); adrianbeznea@hotmail.com (A.B.); george.tocu@ugal.ro (G.Ț.); oanamihailov@yahoo.com (O.M.M.); mihaela.patriciu@ugal.ro (M.P.); liliana.baroiu@ugal.ro (L.B.); 2 Clinical Emergency Hospital “Sf. Ap. Andrei”, 800578 Galați, Romania; pascaru.grigore@yahoo.com; 3 Clinical Children Emergency Hospital “Sf. Ioan”, 800487 Galați, Romania; virginia.lutenco@gmail.com; 4 Clinical Hospital of Infectious Diseases “Sf. Cuvioasa Parascheva”, 800179 Galați, Romania

**Keywords:** peritonitis, prognostic, abdominal sepsis, comorbidities

## Abstract

Generalized secondary peritonitis is a life-threatening intra-abdominal infection requiring urgent surgical intervention. Despite advances in surgical and antimicrobial therapy, morbidity and mortality remain high. Identifying key prognostic factors is crucial for improving patient outcomes. This review examines significant prognostic indicators and explores the potential role of scoring systems and artificial intelligence in risk stratification. A review was conducted using PubMed, Web of Science, Scopus, and Medline databases. Studies published from 2000 to 2024 focusing on prognostic factors in secondary peritonitis were included. A total of 145 studies were identified, with 40 selected based on relevance and methodological quality. Data extraction included patient demographics, comorbidities, severity scores, microbiological profiles, and artificial intelligence applications in peritonitis management. Poor prognosis was associated with advanced age, severe sepsis, organ failure, chronic kidney disease, cardiovascular comorbidities, and diabetes mellitus. The Mannheim Peritonitis Index (MPI) remains a widely validated prognostic tool, while APACHE II and SOFA scores also provide valuable risk estimates. Increasing multidrug-resistant infections further complicate management and impact outcomes. Emerging evidence suggests that machine learning algorithms may improve early risk stratification and individualized outcome prediction when integrated with conventional scoring systems. Identifying prognostic factors remains essential for optimizing outcomes in secondary peritonitis, and future research should prioritize the clinical validation and integration of AI-based models into perioperative management protocols.

## 1. Introduction

Peritonitis is one of the most frequent and severe surgical pathologies, with a significant impact on human health due to its potentially life-threatening nature. It is a condition that occurs in the peritoneal cavity and is characterized by localized or generalized inflammation of the peritoneum. The cause can be bacterial, fungal, or chemical in nature, each with different clinical implications. Peritonitis is generally classified into three main types: primary peritonitis, secondary peritonitis, and tertiary peritonitis.

Primary peritonitis, or spontaneous bacterial peritonitis, is a spontaneous bacterial infection of the peritoneal cavity that occurs most often in the early years of life in children and in adult patients with compromised immunity, such as those with liver cirrhosis. Secondary peritonitis is represented by the secondary inflammation of the peritoneum due to injuries to the intraperitoneal organs, either by perforation, necrosis, or ongoing inflammation. Tertiary peritonitis is a more complex pathology with a slightly more blurred definition, characterized by persistent peritoneal inflammation even after secondary peritonitis has already been treated surgically. It persists for more than 48 h, either because of the increased virulence and antibiotic resistance of the microorganisms involved or due to the host’s immunocompromised state [1].

Peritonitis can also be classified as localized or generalized, depending on the extent of the inflammatory process. In this article, we will focus on secondary peritonitis, given its higher incidence in surgical departments and the near-universal requirement for surgical intervention. The incidence of secondary peritonitis is difficult to assess precisely, given the wide variety of underlying pathologies that may cause it. According to some studies, secondary peritonitis accounts for approximately 1% of all presentations to the hospital emergency department and represents the second leading cause of sepsis in patients admitted to intensive care units.

The first statistical data on the treatment of peritonitis became available at the end of the 19th century, with notable contributions from Mikulicz, Kronlein, and Körte, who published remarkable and pioneering works describing peritonitis [2,3,4]. One of the first surgeons to significantly reduce postoperative mortality in peritonitis was Kirschner, who managed to decrease it from 80–100% to around 60% in 1926 [5]. Despite the tremendous progress in medical imaging, laboratory diagnostics, and the wide array of available antibiotics, the mortality rate in peritonitis remains disturbingly high, particularly in patients who develop severe sepsis, where mortality can range between 35 and 55% [6].

The therapeutic goal in secondary peritonitis is to completely eliminate the septic focus, remove necrotic tissue, perform extensive lavage of the peritoneal cavity, and ensure adequate surgical drainage. Although we now benefit from advanced surgical techniques and intensive care, the overall management of patients with secondary peritonitis has not fundamentally changed since the early modern surgical era. It remains a highly complex and demanding challenge for surgeons.

Certainly, one of the key factors in the unfavorable evolution of secondary peritonitis is the presence of associated patient-related risk factors. The etiology, prevalence, and impact of acute surgical abdomen due to generalized peritonitis are highly variable. Many patients are over the age of 60, with multiple comorbidities and poor general condition, making management more difficult [7]. At present, there is a lack of relevant, comprehensive studies that compile and analyze prognostic factors for generalized secondary peritonitis across different university centers to offer a unified conclusion.

In this review, we aim to explore and identify the most significant prognostic factors influencing the evolution of generalized secondary peritonitis and to highlight those that most strongly correlate with clinical outcomes.

## 2. Materials and Methods

This article was developed with the primary aim of critically analyzing and synthesizing the most relevant and up-to-date knowledge regarding the prognostic factors that influence morbidity and mortality outcomes in cases of generalized secondary peritonitis. To achieve this, a systematic literature search was conducted across four major biomedical databases: PubMed, Web of Science, Scopus, and Medline. These databases were selected due to their extensive coverage of peer-reviewed medical and surgical literature.

We performed a detailed keyword-based search using terms such as peritonitis, prognostic, mortality, morbidity, abdominal sepsis, and artificial intelligence. Boolean operators (AND, OR) were applied to refine and expand the search as appropriate. An example follows: (“*secondary peritonitis*” OR “*abdominal sepsis*”) AND (“*prognostic factors*” OR “*mortality*” OR “*morbidity*”). These keywords were selected to reflect both traditional clinical parameters and modern analytical approaches, including the increasing role of data-driven tools in medical research.

The search was limited to articles published in the English language between 1 January 2000, and 1 March 2024. Additionally, the references of key articles were manually screened to identify other relevant studies not retrieved by the initial search. However, a few select older articles were also included when they provided valuable foundational or historical context regarding the management and understanding of peritonitis.

Despite considering data from the last two decades, special emphasis was placed on articles published within the last 10 years, as these are more likely to reflect current clinical practices, modern surgical techniques, updated definitions, and recent advances in diagnostic and therapeutic modalities. Inclusion criteria were original articles, clinical trials, cohort studies, case–control studies, systematic reviews, and meta-analyses focused on generalized secondary peritonitis; studies evaluating prognostic factors related to morbidity and/or mortality; and articles providing data on established scoring systems (e.g., Mannheim Peritonitis Index, APACHE II, SOFA) or discussing artificial intelligence applications in peritonitis management. Exclusion criteria included case reports, editorials, commentaries, conference abstracts, and non-peer-reviewed publications; studies not available in full-text format; and articles addressing peritonitis of a primary, tertiary, or localized nature, or those involving pediatric-only populations. After applying these criteria, the identified articles underwent a two-step screening process: first by title and abstract review, followed by full-text evaluation to assess their relevance and quality.

## 3. Results

A total of 145 scientific articles were initially identified as potentially relevant to our topic. After screening the titles, 93 publications were selected for further evaluation. Upon reading the abstracts, the number of included studies was narrowed down to 61. In the subsequent step, these 61 articles were analyzed in full, with careful attention to their methodology, relevance, and clinical applicability. Ultimately, 40 of the most pertinent and high-quality articles, in our assessment, were chosen for inclusion in this review. These articles specifically addressed prognostic factors in generalized secondary peritonitis and provided significant insights that supported the objectives of our work.

Data extraction focused on several critical aspects, including patient demographics, pre-existing comorbidities, clinical severity scores, microbiological profiles, and the emerging role of artificial intelligence in the diagnosis, risk stratification, and management of peritonitis. Each of these variables was considered in light of its potential to influence patient outcomes, particularly regarding morbidity and mortality rates.

We aimed to consolidate current knowledge on prognostic factors, both traditional and novel, and examined how widely recognized scoring systems—such as the Mannheim Peritonitis Index (MPI), APACHE II, and SOFA—are currently used in clinical practice. Furthermore, we explored how these scores are evolving and to what extent they can be integrated with advanced computational methods, particularly artificial intelligence and machine learning algorithms, which show promise in enhancing predictive accuracy and guiding personalized treatment strategies. These tools represent a potential paradigm shift in how clinicians may soon approach complex intra-abdominal infections such as generalized secondary peritonitis.

**Figure 1:**
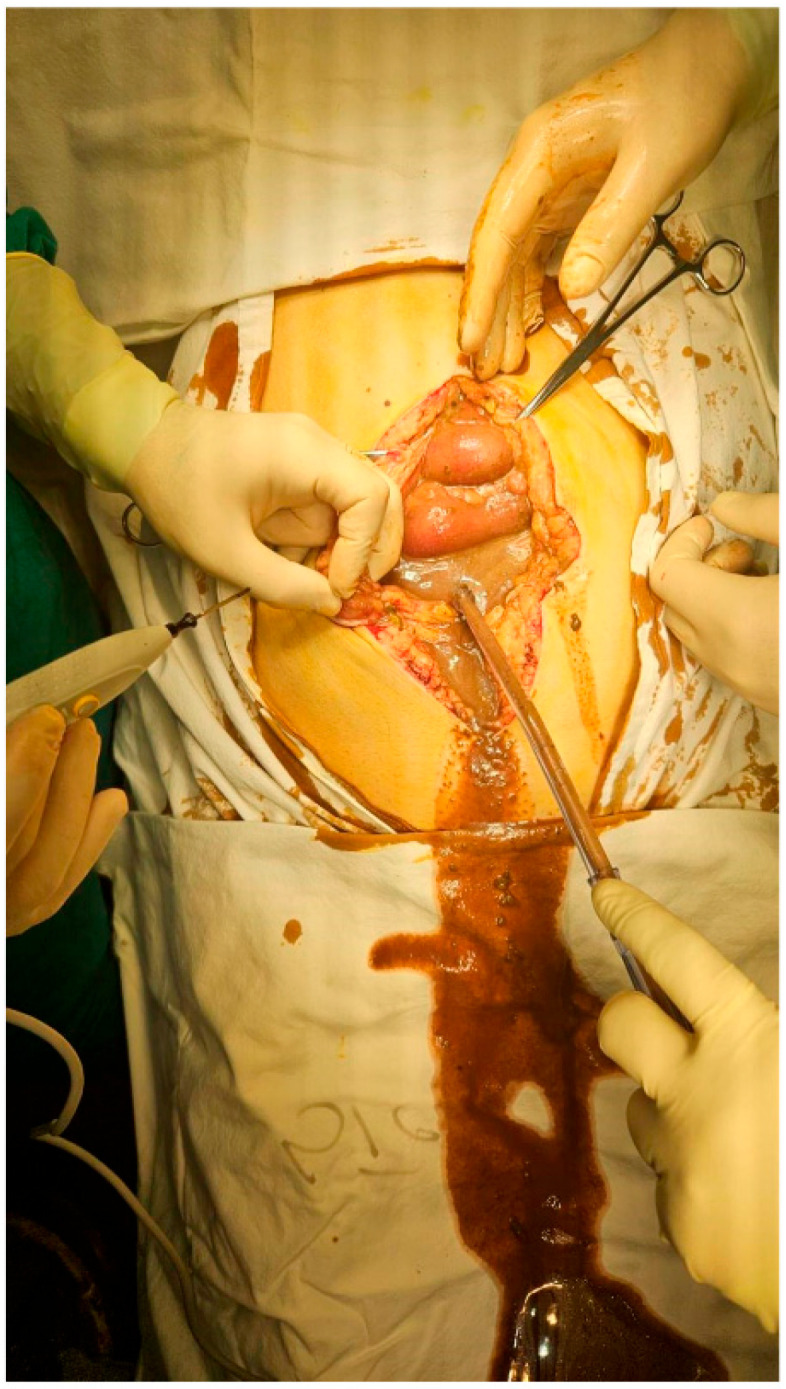
Peritonitis due to the perforation of the colon [10].

Secondary peritonitis is a frequently encountered surgical emergency that poses a serious and life-threatening condition, often associated with elevated rates of mortality and morbidity. Prompt and effective source control, when combined with targeted antibiotic therapy, state-of-the-art intensive care, and modern sepsis management strategies, plays a pivotal role in determining patient outcomes [8]. While intra-abdominal sepsis can affect individuals of all ages, it exerts a disproportionately greater impact on elderly patients compared to their younger counterparts. The clinical presentation typically involves signs and symptoms characteristic of an acute abdomen, which usually allows for a relatively rapid clinical diagnosis of peritonitis. However, a substantial number of patients present to the hospital at a late stage, with already well-established generalized peritonitis, purulent contamination, and varying degrees of systemic involvement and septicemia [Figure 1]. A reduced physiological reserve, together with pre-existing systemic illnesses, contributes to significantly worse outcomes—particularly among the elderly, immunocompromised individuals, and patients with serious or multiple comorbid conditions [9].

### 3.1. Host-Related Aspects

#### 3.1.1. Age

Age is one of the most frequently used demographic variables in medical research and serves as a fundamental parameter in many statistical models and clinical studies. The aging process introduces a range of metabolic, structural, and functional changes in tissues, which amplify the body’s susceptibility to harmful stimuli and reduce its capacity for recovery. These physiological changes significantly increase vulnerability in older patients. In high-income countries, individuals over the age of 60 now constitute a considerable segment of the population, making age an essential and increasingly relevant criterion in patient management and surgical decision-making.

In a study conducted by Alessandro Neri, univariate analysis revealed that “age older than 80 years” was significantly associated with an increased risk of mortality. Furthermore, in multivariate logistic regression, age over 80 remained an independent predictor of mortality [11]. This finding is consistent with another investigation involving 104 patients, which also identified age > 80 years as a statistically significant independent predictor [12]. Similarly, a large-scale study that included 11,202 patients diagnosed with peritonitis demonstrated that advanced age is independently associated with a higher risk of developing severe sepsis in multivariate analysis [13]. 

A number of additional studies have emphasized the prognostic importance of age in secondary peritonitis, identifying age thresholds—often 60 or 70 years—as critical predictors of poor outcomes during univariate analysis. However, it is noteworthy that this significance often diminishes or is lost in multivariate analyses [14,15,16,17]. 

Conversely, other research has suggested that age alone may not have a statistically significant impact on morbidity. In such cases, the observed correlation between age and adverse outcomes is thought to stem more from the presence of comorbidities and diminished physiological reserves rather than age itself [18]. 

Furthermore, age has been confirmed as an independent factor associated with both shock and mortality in patients with peritonitis. It is well known that the incidence of septic shock and the overall mortality rate increase with advancing age, regardless of the infection source [19]. The immunological changes that accompany aging are well documented: reduced chemotaxis and phagocytosis of polymorphonuclear cells, diminished natural killer cell activity, and a general weakening of both the innate and adaptive immune systems. These changes may partly explain the increased susceptibility to infection in older adults. In addition, poor nutritional status and reduced organ reserves—commonly observed in elderly individuals—also contribute to the heightened risk of complications and death [20,21].

#### 3.1.2. Comorbidities

Diffuse peritonitis is a severe and often devastating condition, marked by significant morbidity and mortality. The 30-day mortality rate typically ranges from 15% to 20%, and this rate is notably influenced by the presence of severe comorbid conditions [22,23,24]. The overall health status of the patient is a critical determinant in assessing prognosis and predicting outcomes. To assist clinicians in evaluating patient risk, various scoring systems have been developed. However, these systems often require numerous parameters, making them somewhat cumbersome in urgent surgical settings [25,26].

In practice, surgeons frequently need to rapidly estimate the risk of serious complications or death either preoperatively or intraoperatively, and the time-consuming nature of complex prognostic tools can limit their practical use. As a result, the patient’s perceived prognosis often plays a key role in guiding intraoperative decision-making.

The most commonly reported comorbidities that adversely affect patient outcomes include malignancies, preoperative organ dysfunction, chronic renal insufficiency, cardiovascular conditions, and diabetes mellitus [13,22,27].

A study by Petr Špička, published in 2022 and involving 274 patients with diffuse peritonitis, concluded that having two or more serious comorbidities represents a major negative prognostic factor, significantly increasing both mortality and morbidity rates. In his cohort, 61% of patients had two or more severe comorbidities—a statistic that correlates with the age distribution of his study group. It is evident that older individuals, especially those with multiple concurrent health issues, are more predisposed to developing peritonitis and experiencing worse outcomes. His analysis found that cardiovascular disease, cancer, hypertension, and the presence of multiple severe comorbidities notably raised both morbidity and mortality, which is in line with recent findings in this field. Interestingly, while pulmonary disease was linked to increased mortality, it did not appear to influence morbidity rates in his study [28].

Similarly, in a multivariate logistic regression analysis, Tolonen identified sepsis severity, chronic kidney disease, and pre-existing cardiovascular conditions as independent predictors of 30-day mortality or ICU admission. Moreover, factors such as corticosteroid therapy, metastatic malignancy or lymphoma, and high sepsis severity were also statistically significant predictors of poor outcomes [22].

In a 2013 study by Parwez Sajad Khan, the presence of comorbidities had a profound impact on both morbidity and mortality. Specifically, 58.5% of patients with comorbidities developed complications and 39% died, compared to only 18.6% with complications and 1.6% mortality in patients without any comorbid conditions [18].

The correlation between comorbidities and unfavorable outcomes is strongly supported by numerous studies conducted by other researchers [29,30,31].

#### 3.1.3. Laboratory Biomarkers

Laboratory biomarkers play a crucial role in assessing the prognosis of patients with secondary generalized peritonitis. Elevated serum lactate levels have been consistently associated with increased mortality. For instance, a study by Sahoo demonstrated that preoperative lactate levels exceeding 2.75 mmol/L were predictive of 28-day mortality in patients undergoing emergency laparotomy for perforation peritonitis [32]. Similarly, Sharma et al. found that both preoperative and 24-h postoperative lactate levels were independent predictors of mortality in secondary peritonitis patients [33]. Procalcitonin (PCT) is another biomarker that has shown significant prognostic value. Frigerio and Bassi reported that PCT levels of 10.0 ng/mL or higher on two consecutive days were superior to C-reactive protein (CRP) levels in predicting septic multiorgan dysfunction syndrome (MODS) in secondary peritonitis patients [34]. Additionally, higher PCT levels were associated with increased risk of septic shock and mortality, with cut-off values of 15.3 ng/mL and 19.6 ng/mL, respectively [32]. CRP, while widely used, has shown variable prognostic significance. Elevated CRP levels in the early postoperative phase have been associated with increased mortality in patients operated for severe secondary peritonitis [32]. However, its predictive value may be limited compared to PCT. Hypoalbuminemia has also been identified as an independent predictor of poor outcomes. A study by Lin found that hypoalbuminemia was associated with inferior short-term and long-term survival in cirrhotic patients with spontaneous bacterial peritonitis, regardless of renal function impairment [35]. Furthermore, the neutrophil-to-lymphocyte ratio (NLR) has emerged as a potential prognostic marker. Elevated NLR values have been linked to higher mortality rates and prolonged ICU stays in peritonitis patients [36]. However, its prognostic utility may be enhanced when combined with other markers such as CRP. In summary, integrating these laboratory biomarkers into existing scoring systems or artificial intelligence-driven models may improve risk stratification and guide clinical decision-making in secondary peritonitis.

### 3.2. Systematic Scoring Instruments

Various scoring systems are employed to predict the outcomes in patients with peritonitis. These systems serve as valuable tools for forecasting the prognosis and prioritizing treatment to enhance patient care in cases of peritonitis. One of the most famous and important scores for perforating peritonitis is the Mannheim Peritonitis Index (MPI) [Table 1]. It was developed by Linder et al. in 1987 based on clinical observations and risk factors from 1243 patients with purulent peritonitis to predict mortality in cases of perforation peritonitis [37]. The scoring system includes eight factors, covering demographic, physiological, and disease-specific elements, with a maximum score of 47. In the original study, using a cut-off value of >26, the MPI demonstrated good sensitivity (84%), specificity (79%), and overall accuracy (81%) in identifying patients at higher risk of mortality.

Several studies have demonstrated the effectiveness of the MPI as an independent prognostic scoring system for predicting outcomes in secondary peritonitis. We have compared a few of these studies (Table 2).

Pathak et al., in their recent study (2023) on a group of 235 patients, compared different prognostic scores: Mannheim Peritonitis Index (MPI), the Jabalpur Peritonitis Index, and p-POSSUM. It was a prospective observational cohort study conducted between 2018 and 2020 on patients with secondary non-traumatic peritonitis who underwent laparotomy. The study concluded that the Mannheim Peritonitis Index and p-POSSUM had nearly equivalent performance, with AUCs of 0.757 and 0.756, respectively, indicating fair diagnostic performance. The MPI predicted mortality with a sensitivity of 85% (95% CI 74–92) and a specificity of 58% (95% CI 50–65) at a cut-off of MPI ≥ 27 [38].

A 1994 study, one of the earliest but with a large sample size of 2003 patients, concluded that the Mannheim Peritonitis Index is a simple and reliable tool for assessing risk and classifying patients with peritoneal inflammation (sensitivity—86%, specificity—74%) [39].

In a retrospective analysis of 168 patients from 2015, Piotr Budzyński aimed to evaluate the MPI score to determine the probability of death among the Polish population undergoing surgery for peritonitis. The optimal cut-off point for the MPI was calculated based on ROC analysis. He concluded that the MPI is a simple and reliable tool for predicting mortality in patients undergoing surgery for peritonitis. It also aids in assessing the risk of postoperative complications and determining the need for intensive care unit treatment. Although the Mannheim score is simple to use and effective, it has some disadvantages. It cannot serve as a preoperative system for stratifying patients by death risk at admission. This is because it requires intraoperative evaluation, such as the type of fluid in the peritoneal cavity, the anatomical site of perforation, and histopathological analysis. Another limitation of the score is that it does not account for chronic diseases or major systemic disorders, which are critical risk factors for mortality and serious complications [40].

In 2017, Pattanaik published a prospective observational study, stating that “the ROC curve showed the highest sensitivity and specificity of 79% and 70%, respectively, at an MPI of 25”. He concluded that the MPI system is effective in predicting mortality, easy to use, and an excellent option for predicting morbidity [41]. Other studies have reached similar conclusions about MPI’s predictive capacity but also acknowledged its limitations in contemporary practice [42,43,44,45].

**Table 2:** Comparative analysis of MPI scores (some randomly chosen studies).

Study	Sample Size	Sensitivity (%)	Specificity (%)
Pathak et al. (2023) [39]	235	85	58
Rajesh Sharma et al. (2015) [43]	100	92	78
Muralidhar et al. (2014) [44]	50	72	71
Billing et al. (1994) [40]	2003	86	74
Budzyński et al. (2015) [41]	168	66.7	97.9
S. K. Pattanaik et al. (2017) [42]	120	79	70
Caronna et al. (2021) [45]	70	77.8	72.1

In contrast, p-POSSUM (Physiological and Operative Severity Score for the enumeration of Mortality and Morbidity) is a more comprehensive system that combines physiological parameters and operative severity, making it applicable pre- and intra-operatively. It is, however, more data intensive and less practical in resource-limited settings. The Jabalpur Peritonitis Index, predominantly used in South Asian populations, incorporates factors like age, malignancy, organ failure, and type of peritoneal contamination. While easier to apply, its validation is limited outside its originating regions (Table 3).

Moving forward, next-generation prognostic models should incorporate AI-driven predictive analytics and multidimensional datasets (including genomics, microbiome profiles, and inflammatory biomarkers). Moreover, future tools must be externally validated across diverse populations and healthcare systems and offer real-time prognostic updates as clinical conditions evolve perioperatively.

### 3.3. Microbial Effect

The microbiological landscape of secondary peritonitis is continually evolving, posing new challenges for diagnosis, treatment, and prognosis. Understanding the microbial spectrum is critical, as it directly influences the initial empirical antibiotic strategy, which is a cornerstone in the management of intra-abdominal infections.

Secondary peritonitis is typically polymicrobial, involving a combination of aerobic and anaerobic bacteria. The most frequently isolated organisms include *Escherichia coli*, *Klebsiella pneumoniae*, *Enterococcus* spp., *Bacteroides fragilis*, and *Pseudomonas aeruginosa*. However, recent studies show a worrying trend toward increasing antimicrobial resistance, especially among Gram-negative enteric bacteria and nosocomial pathogens.

The “Complicated Intra-Abdominal Infections Worldwide Observational Study” (CIAOW) highlighted a global rise in extended-spectrum beta-lactamase (ESBL)-producing organisms, particularly *E. coli*, whose prevalence nearly tripled from 2002 to 2008 [46,47]. Resistance to *Klebsiella pneumoniae* is also on the rise, with up to 20% of strains showing resistance to carbapenems in some regions. The emergence of carbapenem-resistant Enterobacteriaceae (CRE) has become an especially serious threat, limiting treatment options and increasing mortality risk.

In addition to Gram-negative organisms, *Enterococcus* species—which are typically resistant to many antibiotics—have become more commonly isolated in hospital-acquired infections. Their clinical relevance is debated, but increasing evidence suggests that Enterococci, especially vancomycin-resistant strains (VRE), are associated with worse outcomes in critically ill patients.

*Pseudomonas aeruginosa* represents another concern due to its intrinsic resistance mechanisms and ability to acquire new resistance rapidly. Multiple studies have confirmed that *Pseudomonas* infections are independent risk factors for mortality in patients with abdominal sepsis [48].

Fungal infections, particularly *Candida* species, are gaining more attention as well. These infections are usually seen in critically ill patients, especially those with prolonged antibiotic use, immunosuppression, or gastrointestinal perforations. Candidal peritonitis is associated with significantly increased morbidity and mortality, with some studies reporting mortality rates exceeding 50% in patients with candidemia following abdominal surgery [49,50].

Importantly, antimicrobial resistance and microbial profiles display significant regional variability, influencing both prognosis and management. In high-income countries, the widespread use of broad-spectrum antibiotics and advanced infection control measures have led to different resistance patterns compared to low- and middle-income countries (LMICs), where antibiotic misuse and limited surveillance contribute to higher rates of multidrug-resistant organisms (MDROs). For example, studies from Southeast Asia and parts of Africa report alarmingly high rates of ESBL-producing Enterobacteriaceae and carbapenem-resistant strains, directly impacting mortality rates and length of hospital stay. In contrast, data from Northern Europe and parts of North America indicate relatively lower prevalence of CRE but rising incidences of VRE and drug-resistant Pseudomonas.

The prognostic implications of these variations are profound. In regions with high MDRO prevalence, delayed appropriate therapy and limited effective antimicrobials have been associated with higher mortality and complication rates in peritonitis patients. As a result, empirical therapy protocols must be tailored not only to hospital-specific antibiograms but also to regional resistance patterns to optimize patient outcomes.

Surgeons and intensivists must also be alert to the geographical variations in microbial patterns and resistance. In low- and middle-income countries, the misuse and overuse of antibiotics contribute heavily to resistance, emphasizing the need for global and coordinated action. For this reason, we support the initiatives of the Global Alliance for Infections in Surgery, which aims to educate healthcare providers and promote best practices in surgical infection management [51].

Furthermore, there is growing interest in integrating molecular diagnostic techniques, such as PCR and next-generation sequencing, into clinical workflows. These methods can detect pathogens more rapidly than traditional cultures and may become vital tools in early pathogen identification, especially in critically ill patients where time is a limiting factor.

To improve prognostic evaluation further, future studies should explore integrating regional microbiological profiles and resistance data into existing prognostic scoring systems or AI-driven models. This could help refine risk stratification and clinical decision-making in both high-resource and resource-limited settings.

### 3.4. Future Directions: Artificial Intelligence

Artificial intelligence (AI) is no longer a concept of the future—it is rapidly becoming an integral part of modern medicine, including the management of complex surgical emergencies such as generalized secondary peritonitis. Terms like AI, machine learning (ML), and deep learning (DL) are increasingly prevalent, representing powerful tools for improving clinical decision-making, especially in time-sensitive conditions such as intra-abdominal sepsis [52].

The management of peritonitis involves interpreting vast amounts of data—ranging from clinical signs and laboratory results to radiological findings and operative details. AI can process and analyze these multidimensional datasets far beyond human capacity, revealing hidden patterns, correlations, and predictive variables that may not be readily apparent using traditional methods. This capability is particularly promising in identifying high-risk patients and predicting morbidity and mortality.

Currently, several types of AI models have been explored in the context of intra-abdominal infections and sepsis, including logistic regression, decision trees, random forests, support vector machines, and more recently, deep learning frameworks such as convolutional neural networks (CNNs) for image analysis and recurrent neural networks (RNNs) for time series clinical data. These models typically incorporate variables such as patient age, comorbidities, inflammatory markers (e.g., CRP, procalcitonin), organ dysfunction scores (SOFA, APACHE II), intraoperative findings, and microbiological data. In comparative studies, AI-based models have demonstrated superior predictive accuracy over conventional scores; for instance, a 2022 multicenter study reported an area under the curve (AUC) of 0.89 for an AI-driven mortality prediction model in abdominal sepsis, outperforming the SOFA score (AUC 0.78) and APACHE II (AUC 0.76). Integrating such specific examples underscores AI’s tangible progress and moves the conversation from theoretical promise to practical, evidence-based applications [53]. For example, predictive algorithms enhanced with AI may allow for real-time mortality risk estimation, enabling more precise triage, earlier intensive care admission, or tailored surgical planning.

AI-enhanced imaging is another exciting area. Deep learning algorithms trained on large datasets of radiological images can now detect subtle abnormalities in CT scans or X-rays—such as free air, localized abscesses, or bowel wall thickening—faster and with greater consistency than human readers. This could lead to earlier detection of perforation or abscess formation, potentially reducing time to intervention and improving prognosis [54].

Beyond diagnostics, AI also plays a growing role in therapeutic optimization. It can help identify ideal antibiotic regimens by analyzing microbial patterns and resistance trends in real time. Additionally, AI can suggest treatment modifications based on predicted patient response, drug interactions, and comorbidity profiles. This personalized approach may reduce overtreatment, limit resistance, and lower healthcare costs.

While many AI applications in peritonitis management are still under investigation, several have already been integrated into clinical practice. For instance, a study by Cheng et al. (2023) implemented an AI-powered LINE Chatbot to enhance self-care among peritoneal dialysis patients, resulting in a reduction of peritonitis incidence from 0.93 to 0.8 episodes per 100 patient-months [55]. Additionally, AI-enhanced imaging tools utilizing deep learning algorithms have been employed to detect subtle abnormalities in CT scans, such as free air or localized abscesses, facilitating earlier diagnosis and intervention. These examples demonstrate that AI is not solely a future prospect but is currently augmenting clinical decision-making in specific contexts.

Another study from 2023 explored AI in the diagnosis of acute appendicitis, including cases with delayed presentations resulting in secondary peritonitis. The researchers found that AI algorithms surpassed traditional clinical tools, such as the Alvarado score, in both diagnostic speed and accuracy—demonstrating AI’s versatility across multiple abdominal emergencies [56].

Despite these promising developments, several challenges and limitations must be addressed before AI can be fully integrated into routine clinical practice. The quality and consistency of input data remain critical concerns; AI algorithms require access to high-quality, well-annotated, and comprehensive datasets to ensure reliable outputs. In many cases, existing medical records may suffer from incomplete, inconsistent, or biased documentation, which can compromise model performance.

Additionally, the composition of algorithm training samples greatly influences AI accuracy and generalizability. If training datasets lack diversity in terms of patient demographics, comorbidities, or institutional protocols, the resulting models may exhibit reduced performance when applied to different patient populations or healthcare settings. This issue emphasizes the importance of cross-institutional data integration and collaboration, which would allow AI systems to be trained and validated on large, diverse, multicenter datasets, thereby improving their robustness and external validity.

Moreover, the regulatory landscape and ethical considerations must evolve in parallel with technological advances. Establishing clear frameworks for AI validation, approval, and post-implementation monitoring is essential to ensure safety, transparency, and reproducibility. Concerns about data privacy, algorithmic bias, and the potential erosion of clinician autonomy must also be carefully managed. AI should not replace human expertise but instead augment decision-making, empowering clinicians with faster, more reliable tools.

While the potential of artificial intelligence in managing generalized secondary peritonitis is substantial, it is important to recognize that many AI-driven applications currently remain within the research and developmental stages. Although promising real-world examples—such as AI-enhanced imaging analysis and decision support tools for peritoneal dialysis care—are emerging, widespread clinical implementation is still limited. Most predictive models and therapeutic optimization systems require further validation through large, multicenter studies before they can be reliably adopted into everyday practice. As such, AI presently serves as a complementary tool in select areas, with its broader integration into clinical workflows anticipated in the near future as technological, regulatory, and infrastructural challenges are addressed.

For instance, a study utilizing the Japanese Nationwide Clinical Database developed predictive models for surgical outcomes in acute diffuse peritonitis (ADP). These models incorporated variables such as patient age, cancer metastasis, platelet count, serum albumin levels, and the anatomical site of peritonitis. The models demonstrated high predictive accuracy, with C-indices of 0.859 for perioperative mortality and 0.857 for 30-day mortality, suggesting their potential utility in clinical settings [57].

In conclusion, artificial intelligence represents a transformative force in the future of peritonitis care. With continued research, interdisciplinary collaboration, and ethical implementation, AI has the potential to revolutionize diagnosis, prognostication, and therapy in patients with generalized secondary peritonitis.

### 3.5. Limitations of the Study

This review is subject to several limitations. First, the selection of articles was based on subjective judgment regarding relevance, which may have introduced selection bias. Although we used multiple databases and systematic criteria, non-English studies and gray literature were excluded, potentially limiting the breadth of available data. Additionally, many of the included studies were retrospective in nature or based on single-center experiences, which may affect the generalizability of their findings. The integration of artificial intelligence in clinical settings is still in early stages, and the current literature often lacks standardized methodologies or long-term outcome data.

### 3.6. Implications for Clinical Practice

Understanding the prognostic factors in generalized secondary peritonitis is critical for timely risk stratification and management. The recognition of age, comorbidities, and severity scores such as the MPI can support surgeons and intensivists in making informed decisions. Furthermore, the growing role of artificial intelligence in healthcare suggests a shift toward data-driven, personalized medicine. As predictive models evolve, incorporating AI tools could enhance early diagnosis, improve resource allocation, and ultimately reduce morbidity and mortality in this high-risk population. However, when it comes to secondary generalized peritonitis specifically, practical applications of AI remain scarce. Current studies in this area are primarily experimental, with most confined to retrospective analyses or theoretical model development without clinical implementation. No AI-driven models have yet been validated for routine use in managing secondary peritonitis, and available results remain preliminary. While AI holds potential for enhancing decision-making and risk assessment in the future, further prospective, disease-specific research is necessary to translate these technologies into practical tools for clinicians managing secondary peritonitis.

## 4. Conclusions

Generalized secondary peritonitis remains a challenging surgical emergency with a multifactorial prognosis. While a vast amount of data exists regarding prognostic factors, synthesizing this information into a practical, systematic framework remains complex. However, certain variables consistently emerge across studies as key indicators of poor outcomes. Advanced age, severe sepsis, active malignancy, preoperative organ failure, chronic kidney disease, cardiovascular conditions, and diabetes mellitus have all been identified as significant contributors to increased morbidity and mortality. Laboratory biomarkers also have shown prognostic relevance in secondary peritonitis, and their integration into clinical assessment may enhance the accuracy of outcome prediction

To better assess prognosis and guide clinical decision-making, several scoring systems have been developed—among which the Mannheim Peritonitis Index (MPI) stands out for its simplicity, reproducibility, and consistent performance in various populations. Nevertheless, even the best traditional models have limitations, particularly in their ability to adapt to individual patient nuances or evolving clinical patterns.

In this context, artificial intelligence (AI) offers exciting potential. By leveraging large, heterogeneous datasets—including demographic, clinical, microbiological, and radiological data—AI has the capacity to enhance existing prognostic tools, uncover new predictive patterns, and support more precise, personalized patient care. Looking forward, future research should focus on the development and validation of dynamic, AI-enhanced prognostic models that incorporate patient-specific clinical, microbiological, and regional epidemiological data. Additionally, multinational collaborations are needed to address data disparities between high-income and resource-limited settings, ensuring that prognostic tools remain globally applicable and equitable.

In conclusion, while traditional prognostic factors and scoring systems continue to offer valuable guidance, the integration of AI-driven approaches represents the next frontier in improving outcomes for patients with generalized secondary peritonitis. This study highlights both the current achievements and the critical gaps that future research must address to enhance prognostic precision and optimize patient care.

## Figures and Tables

**Table 1:** Mannheim Peritonitis Index (MPI).

Risk Factor	Score
Age > 50 years	5
Female sex	5
Organ failure	7
Malignancy	4
Preoperative duration of peritonitis > 24 h	4
Non-colonic origin of sepsis	4
Diffuse generalized peritonitis	6
**Exudate**	
Clear	0
Cloudy, purulent	6
Fecal	12
**Definitions of organ failure**	
Kidney	Creatinine level > 177 μmol/L Urea level > 167 mmol/L Oliguria < 20 mL/h
Lung	PO₂ < 50 mmHg PCO₂ > 50 mmHg
Shock	Hypodynamic or hyperdynamic

**Table 3:** Comparative performance and characteristics of common prognostic scoring systems in secondary peritonitis.

Scoring System	Key Features	Required Parameters	Application Timing	Reported Sensitivity	Reported Specificity	Advantages	Limitations
**Mannheim Peritonitis Index (MPI)**	Clinical/intraoperative factors	Age, malignancy, organ failure, origin of sepsis, contamination type, etc.	Intraoperative	79–86% [37,38,39,40,41]	58–79% [37,38,39,40,41]	Simple, globally validated	Requires intraoperative findings; excludes comorbidities
**p-POSSUM**	Physiological + operative severity factors	Pre-op vitals, blood tests, intra-op findings	Pre- and intraoperative	80–90% (varies by study)	70–85%	High accuracy, preoperative utility	Data intensive, requires extensive parameters
**Jabalpur** **Peritonitis Index (JPI)**	Age, organ failure, malignancy, contamination type	Limited clinical and intra-op data	Intraoperative	75–85% (South Asian cohorts)	60–80%	Simple, cost-effective	Limited validation outside South Asia
**AI-based Models (Emerging)**	Multivariate algorithms incorporating demographics, labs, imaging, comorbidities	Variable (depending on model)	Pre-, intra-, and postoperative	85–95% (preliminary studies)	80–90%	Real-time, adaptable, integrates big data	Requires high-quality data, limited clinical integration
